# Effect of Text Messaging on Bowel Preparation and Appointment Attendance for Outpatient Colonoscopy: A Randomized Clinical Trial

**DOI:** 10.1001/jamanetworkopen.2020.34553

**Published:** 2021-01-25

**Authors:** Nadim Mahmud, David A. Asch, Jessica Sung, Catherine Reitz, Mary S. Coniglio, Caitlin McDonald, Donna Bernard, Shivan J. Mehta

**Affiliations:** 1 Division of Gastroenterology and Hepatology, Perelman School of Medicine, University of Pennsylvania, Philadelphia; 2 Leonard David Institute of Health Economics, University of Pennsylvania, Philadelphia; 3 Center for Clinical Epidemiology and Biostatistics, Department of Biostatistics, Epidemiology & Informatics, Perelman School of Medicine, University of Pennsylvania, Philadelphia; 4 Corporal Michael J. Crescenz VA Medical Center, Philadelphia, Pennsylvania; 5 Center for Health Care Innovation, University of Pennsylvania, Philadelphia

## Abstract

**Importance:**

Outpatient colonoscopy is important for colorectal cancer screening. However, nonadherence and poor bowel preparation are common.

**Objective:**

To determine if an automated text messaging intervention with a focus on informational and reminder functions could improve attendance rates and bowel preparation quality for outpatient colonoscopy.

**Design, Setting, and Participants:**

This randomized clinical trial was conducted in an endoscopy center at an urban academic medical center. Adult patients scheduled for outpatient colonoscopy between January and September 2019 were enrolled by telephone call (early phase) or by automated text message (late phase). Data were analyzed from October 2019 to January 2020.

**Interventions:**

After enrollment, patients were randomized in a 1:1 ratio to usual care (ie, written instructions and nurse telephone call) or to the intervention (ie, usual care plus an automated series of 9 educational or reminder text messages in the week prior to scheduled colonoscopy).

**Main Outcomes and Measures:**

The primary outcome was appointment attendance rate with good or excellent bowel preparation. Secondary outcomes included appointment attendance rate, bowel preparation quality (poor or inadequate, fair or adequate, and good or excellent), and cancellation lead time (in days).

**Results:**

Among 753 patients included and randomized in the trial (median [interquartile range] age, 56 [49-64] years; 364 [48.3%] men; 429 [57.2%] Black), 367 patients were randomized to the intervention group and 386 patients were randomized to the control group. There was no significant difference in the primary outcome between groups (patients attending appointments with good or excellent bowel preparation: intervention, 195 patients [53.1%]; control, 210 patients [54.4%]; *P* = .73), including when stratified by early or late phase enrollment groups. Similarly, there were no significant differences in secondary outcomes.

**Conclusions and Relevance:**

This randomized clinical trial found no significant difference in appointment attendance or bowel preparation quality with an automated text messaging intervention compared with the usual care control. Future work could optimize the content and delivery of text message interventions or identify patient subgroups that may benefit from this approach.

**Trial Registration:**

ClinicalTrials.gov Identifier: NCT03710213

## Key Points

**Question:**

Do automated text messages improve outpatient colonoscopy attendance rate and bowel preparation quality?

**Findings:**

In this randomized clinical trial including 753 patients, usual care patient instructions were compared with an automated text messaging intervention in the week prior to outpatient colonoscopy. There was no significant difference between groups in appointment attendance rate or bowel preparation quality.

**Meaning:**

This randomized clinical trial found that automated text message reminders and instructions did not improve outpatient colonoscopy adherence, although future studies may identify patient subgroups that benefit from this approach.

## Teaser

This randomized clinical trial examines the effectiveness of information and reminders via automated text message vs usual care for coloscopy attendance and preparation.

## Introduction

Colorectal cancer is the second leading cause of cancer-related death in the United States,^1^ yet there are effective screening and treatment strategies that allow for early detection and treatment.^2^ Colorectal cancer screening, which could include stool testing or colonoscopy, is recommended for all individuals aged 50 to 75 years, but national rates remain suboptimal.^3^ Colonoscopy is an essential component of colorectal cancer screening, as it is required if stool test results are positive for anomalous DNA changes or blood, and colonoscopy can achieve effective cancer prevention through polypectomy.^4^ However, colonoscopy entails a complex process for patients to identify an escort, obtain and purchase the bowel preparation, take a day off from work, adhere to a clear liquid diet, and complete the preparation as recommended. These challenges contribute to high nonattendance and cancellation rates^5,6^ and poor preparation quality,^7,8^ both limiting the patient and population benefit.

Common approaches to engage patients and improve screening adherence include having nurses or trained health care staff call patients before the procedure.^9^ However, it is often difficult to reliably reach patients on the phone, and the high cost of these interventions put them out of reach of many practices.^10^ Other interventions, such as videos or mobile applications, have been limited by poor user experience or limited engagement with the patient. There is an opportunity to leverage an automated text message navigation intervention to improve patient engagement prior to colonoscopy completion.

We conducted a quality improvement pilot study that evaluated the feasibility of a 1-week text messaging protocol for patients who were scheduled for outpatient colonoscopy.^11^ Among 21 patients enrolled in the pilot study, there was high user acceptability and higher colonoscopy attendance rates compared with baseline values at an urban academic endoscopy center. The aim of this study was to evaluate the effect of an automated text message–based intervention on appointment adherence and bowel preparation quality in a larger, randomized setting.

## Methods

### Study Design

**Figure 1.**
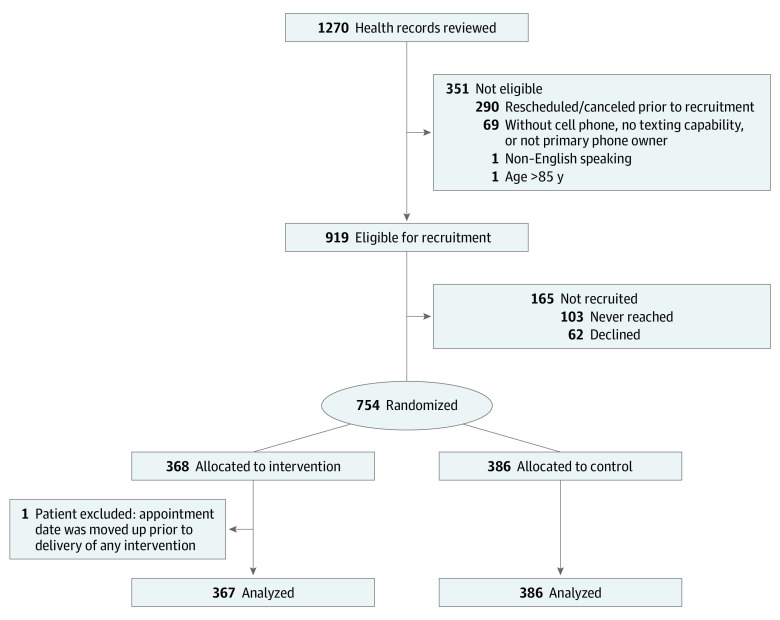
Patient Recruitment Flowchart

This was a pragmatic (ie, conducted in routine clinical practice with minimal exclusions) randomized clinical trial evaluating if an automated text messaging navigation program increased attendance of colonoscopy with adequate bowel preparation compared with usual care. This study received institutional review board approval from the University of Pennsylvania. A waiver of informed consent was obtained, as the study was minimal risk and could not have practicably been carried out without the waiver. The Trial Protocol and Statistical Analysis Plan appear in Supplement 1. This study followed the Consolidated Standards of Reporting Trials (CONSORT) reporting guideline, including the flowchart to track participants during enrollment and trial procedures (Figure 1).

### Study Population and Study Setting

Patients ages 18 to 85 years scheduled for outpatient colonoscopy at the Penn Presbyterian Medical Center an urban medical center located in West Philadelphia, Pennsylvania, who had a text messaging–capable cell phone were eligible for enrollment. This included patients scheduled for colonoscopy for any indication with any of 7 practicing endoscopists. The outpatient endoscopy center accepts open access colonoscopies, for which patients do not require gastroenterology consultation prior to scheduling a colonoscopy. Exclusion criteria included colonoscopy scheduled fewer than 14 days from potential enrollment to allow for adequate time for randomization and setup prior to start of the intervention. Patients were also excluded if they did not speak English and required a translator, were not the primary individual receiving messages on their cell phone, or if they refused participation.

### Recruitment and Randomization

Patients were enrolled in 2 phases between November 2018 and March 2019. In the early phase, 250 patients were enrolled through telephone calls from a clinical research coordinator, using an established script (Trial Protocol in Supplement 1). Using a random number generator, patients were sequentially randomly assigned to 2 arms in a 1:1 ratio to usual care control or usual care plus a text message–based intervention. Usual care consisted of written bowel preparation instructions, and a telephone call and/or an automated voice recording call (Televox) from the endoscopy staff in the week prior to colonoscopy. Patients were also given the endoscopy telephone number and could call to speak to staff if they had specific questions or needed to reschedule. A standard split-dose bowel preparation regimen with polyethylene glycol (MiraLAX [Bayer] and Gatorade [PepsiCo]) and bisacodyl (Dulcolax [Sanofi]) was the default recommendation for all patients, though individual clinicians could prescribe an alternate regimen at their discretion. In the late phase, 500 patients were automatically enrolled using verified cell phone numbers from the electronic health record (EHR) system. Patients were randomized in a 1:1 ratio in variable blocks of 8 and 4. Patients in the intervention arm received 2 enrollment text messages explaining the texting program and providing the opportunity to opt out (by replying with the text *STOP*). Endoscopists were blinded to participant allocation. Of note, 2 phases of enrollment were created to help determine whether the mechanism of enrollment (ie, via telephone vs automated text message) impacted outcomes, because the recruitment via automated text message would be more efficient at scale.

### Intervention

The planned intervention was a series of automated text messages sent to patients in the week prior to scheduled colonoscopy. Messages were sent using the Way to Health platform, an application designed to enhance patient engagement in health care through automated communication.^12^ Way to Health is not linked to the EHR system and requires manual input of a patient identifier and phone number to register patients and schedule automated text messages according to a prespecified intervention protocol. In this study, the Way to Health intervention consisted of scheduled, daily text messages designed to provide educational information in a timely fashion (Table 1). Web links to online bowel preparation instructions were provided, as well as a map link to the endoscopy center. Patients were sent messages at times when dietary changes or laxative consumption were required per protocol. Importantly, the messaging content was designed initially on the basis of existing instructional information but adapted using principles of behavioral science that were tested in a prior pilot study of this intervention.^11^ An important distinction from the intervention in the prior pilot study is that the intervention used in this randomized clinical trial supported only unidirectional (push) messages; there was no bidirectional text communication.

**Table 1.** Intervention Text Messaging Schedule and Content

Timing	Message
At enrollment	Congrats [name] on scheduling your colonoscopy on [date] with [clinician]! We would like to offer you a texting program to guide you through the process. Just so you have it, here is an online link to the prep instructions: [link]
Prior to appointment, d (time)	
7 (8 am)	Hi [name], only one week before your colonoscopy! We will be in touch this week to guide you through the process. If you take any blood thinners (like warfarin), make sure you have discussed this with your prescribing doctor. Some patients may need to stop these before the procedure.
6 (8 am)	Hello, [name]! Hopefully you received a paper copy of your colonoscopy prep instructions. Just in case, here is a link to the same instructions online: [link]
5 (8 am)	Hello [name], don’t forget to pick up your prep materials from the local pharmacy, which includes MiraLAX, Dulcolax, and Gatorade (or Crystal Light or Pedialyte if you have diabetes). This info is in the instructions at this link: [link]
4 (8 am)	Good morning, [name]! Only 4 d to go until your procedure! If you have any questions about the colonoscopy, please call 215-662-9131 between 8 am and 6 pm.
3 (8 am)	Hi [name], make sure you have someone to take you home after your colonoscopy. Plan to arrive one hour before your procedure time. Penn Endoscopy is located at the CUPP Building, 51 N. 39th St., Phila. PA. Here is a link: [link]
2 (8 am)	[Name], your colonoscopy is in 2 d! Today, avoid high-fiber foods like fruits, vegetables, and seeds. Starting tomorrow morning, you should have only clear liquids until your procedure is complete.
1 (8 am)	[Name], you have already come so far! Continue a clear liquid diet today (liquids you can see through with light colors). Remember, no solid foods! The next step will be to take 4 Dulcolax pills at 4 pm.
1 (4 pm)	It is time to take the 4 Dulcolax pills and mix the MiraLAX with the Gatorade (or Crystal Light or Pedialyte). At 5 pm, you should start drinking the first half gallon of your prep. If you feel nauseated, you can always slow down to help tolerate it. You can do it!
1 (6 pm)	Good job! Start drinking the second half gallon of the prep 6 h before your scheduled arrival time. Try to drink every last drop to get the best prep you possibly can!

### Study Outcomes

The primary outcome was colonoscopy attendance rate with good or excellent bowel preparation (binary outcome). Secondary outcomes included colonoscopy attendance rate (ie, attending the originally scheduled appointment), bowel preparation quality (classified as poor or inadequate, fair or adequate, and good or excellent), colonoscopy cancellation rate, cancellation lead time in days, colonoscopy reschedule rate (ie, canceled but rescheduled at the same time), and colonoscopy appointment nonattendance rate. We also reported the proportion of patients opting out of the intervention in the late phase of enrollment. Of note, bowel preparation quality was assessed subjectively by the endoscopist using the Aronchick scale,^13^ as the Boston Bowel Preparation Scale (BBPS) is not routinely used at our institution.

### Statistical Analysis

We targeted an enrollment of 750 patients, which would provide 80% power to detect a 10% difference in the primary outcome with α = .05. After screening and enrollment based on eligibility criteria, a total of 754 patients were randomized to the intervention and control groups; however, 1 patient was excluded from analysis because the appointment date was moved up prior to delivery of any intervention (Figure 1). Descriptive statistics were computed as medians and interquartile ranges (IQRs) for continuous variables and as numbers and percentages for categorical variables. We compared baseline demographic characteristics and major medical comorbidities between study groups, as well as primary and secondary outcomes using the Wilcoxon rank-sum for continuous data and χ^2^ tests for categorical data. To determine if recruitment strategy impacted outcomes, we additionally compared outcomes between early and late phases of enrollment. Next, to adjust analyses for residual demographic characteristic or comorbidity differences between groups after randomization, we used multivariable logistic regression. Two of us (J. S. and C. M.) performed manual collection of patient demographic characteristics (ie, age, sex, race/ethnicity), comorbidities (ie, diabetes, hypertension, and chronic kidney disease), and opiate use from EHRs. Among patients who attended their colonoscopy appointment, we also collected data on the indications for colonoscopy. Finally, to evaluate for possible subgroups for whom the impact of the intervention might be more or less effective, we tested for several potential interactions in logistic regression models, including those between treatment group and age quartile, sex, race/ethnicity, and opiate use. The threshold for statistical significance was set at 2-sided α = .05. Data were analyzed using Stata statistical software version 15.1/IC (StataCorp). Analyses were conducted from October 2019 to January 2020.

## Results

### Patient Characteristics

Among 753 patients enrolled, 364 participants (48.3%) were men, 429 participants (57.2%) were Black, and 376 participants (49.9%) privately insured; 367 patients were randomized to the intervention group and 386 patients were randomized to the control group. Patients were similar across most demographic and comorbidity variables (Table 2). Among patients who attended their colonoscopy appointment, most colonoscopies were performed for cancer screening or surveillance indications (356 colonoscopies [70.6%]), followed by abnormal bowel habits (51 colonoscopies [10.1%]), gastrointestinal bleeding or anemia (41 colonoscopies [8.1%]), and follow-up of inflammatory bowel disease (40 colonoscopies [7.9%]).

**Table 2.** Patient Characteristics by Study Group

Characteristic	Patients, No. (%)	
	Intervention (n = 367)	Control (n = 386)
Age, median (IQR), y	56 (50-64)	56 (49-64)
Sex		
Men	193 (52.6)	171 (44.3)
Women	174 (47.4)	215 (55.7)
Race/ethnicity		
White	134 (36.5)	136 (35.5)
Black	202 (55.0)	227 (59.3)
Hispanic	0	1 (0.3)
Asian	15 (4.1)	11 (2.9)
Other	16 (4.4)	8 (2.1)
Insurance type		
Medicare	87 (23.7)	99 (25.6)
Medicaid	81 (22.1)	87 (22.5)
Private	184 (50.1)	192 (49.7)
Other	34 (9.3)	26 (6.7)
BMI, median (IQR)	28.4 (24.3-34.2)	28.6 (24.8-34.2)
Comorbidity		
Hypertension	164 (44.7)	163 (42.2)
Hyperlipidemia	87 (23.8)	96 (24.9)
Diabetes	60 (16.4)	63 (16.4)
Congestive heart failure	12 (3.3)	9 (2.3)
Using opioids	50 (13.6)	30 (7.8)
Additional reminders		
None	40 (10.9)	48 (12.4)
Staff call	41 (11.2)	29 (7.5)
Automatic recording	76 (20.7)	85 (22.0)
Staff call and automated recording	210 (57.2)	224 (58.0)

Abbreviations: BMI, body mass index (calculated as weight in kilograms divided by height in meters squared); IQR, interquartile range.

### Outcomes

**Figure 2.**
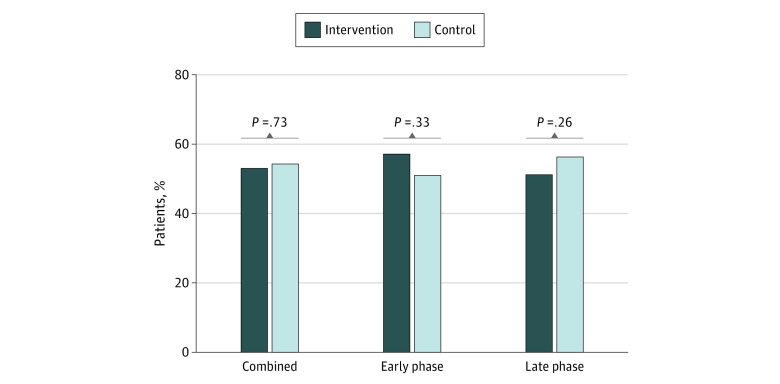
Primary Outcome of Appointment Attendance With Good or Excellent Bowel Preparation

Achievement of the primary outcome, appointment attendance with good or excellent bowel preparation, was similar in the intervention and control groups (195 patients [53.1%] vs 210 patients [54.4%]; *P* = .73) (Figure 2). There were similarly no differences in primary outcome between intervention and control groups when stratified by early phase enrollment (67 patients [57.3%] vs 69 patients [51.1%]; *P* = .33) or late-phase enrollment (128 patients [51.2%] vs 141 patients [56.2%]; *P* = .26) (Figure 2). Secondary outcomes are summarized in Table 3. There were no significant differences between intervention and control groups in in appointment attendance rate (242 patients [65.9%] vs 262 patients [67.9%]; *P* = .57), bowel preparation quality (excellent or good: 195 patients [81.6%] vs 210 patients [82.7%]; fair or adequate: 31 patients [13.0%] vs 28 patients [11.0%]; poor or inadequate: 13 patients [5.4%] vs 16 patients [6.3%]; *P* = .75), or appointment reschedule rate (42 patients [11.4%] vs 51 patients [13.2%]; *P* = .46). Finally, median (IQR) cancellation lead time prior to scheduled colonoscopy was 4 (1-7) days for both groups (*P* = .81). For all secondary outcomes, when stratified by early or late phase enrollment, there remained no significant difference between arms. Similarly, adjustment for differences in sex and baseline opiate use in logistic regression models did not alter the results, and no significant interactions were identified.

**Table 3.** Outcomes by Study Group

Outcome	Patients, No. (%)		*P* value
	Intervention (n = 367)	Control (n = 386)	
Appointment attendance with excellent or good preparation quality	195 (53.1)	210 (54.4)	.73
Appointment attendance	242 (65.9)	262 (67.9)	.57
Bowel preparation quality^a^			
Excellent or good	195 (81.6)	210 (82.7)	.75
Fair or adequate	31 (13.0)	28 (11.0)	
Poor or inadequate	13 (5.4)	16 (6.3)	
Appointment			
Nonattendance	49 (13.4)	50 (13.0)	.87
Cancelled	34 (9.3)	23 (6.0)	.09
Rescheduled	42 (11.4)	51 (13.2)	.46
Cancellation lead time, median (IQR), d	4 (1-7)	4 (1-7)	.81

Abbreviation: IQR, interquartile range.

^a^ Some patients attended their colonoscopy appointment but did not receive the procedure for reasons unrelated to bowel preparation (eg, anticoagulation).

## Discussion

In this randomized clinical trial of patients scheduled for outpatient colonoscopy, we tested the effectiveness of a text message–based intervention in improving appointment attendance rates with good or excellent bowel preparation. We did not find significant differences between this intervention and usual care. Furthermore, among a series of secondary outcomes, including cancellations, nonattendance, appointment rescheduling, and bowel preparation quality, we similarly did not identify any significant differences attributable to the texting intervention.

The results of randomized clinical trials often do not confirm pilot findings; in the pilot study^11^ for this trial, we observed a significant increase in appointment adherence among patients receiving a texting intervention. There may be several reasons for this discrepancy, some of which represent a type of generalizability bias that may occur in transition from pilot to randomized clinical trial.^14^ First, while the text message content in our pilot study was near-identical to that used in this trial, an added feature of bidirectionality was used. This allowed patients to text questions to the Way to Health platform, and these would be answered by study staff within 24 hours. This feature was not felt to be practical in a larger trial or at scale, primarily owing to the need to train all endoscopy nursing staff in the use of Way to Health and reinforce behaviors to check a non-EHR platform regularly, as well as respond to messages over the weekend. Future studies may seek to reevaluate the added value of bidirectional text messages in this context as the conversational nature may provide greater utility and engagement for the patient. Second, convenience sampling to find willing participants in the pilot may have led to a volunteer bias, in which patients receiving the intervention were potentially more engaged in their own health care at baseline. This could have resulted in the pilot intervention falsely appearing to confer benefit. Third, intervention intensity bias likely played a role in the quality of the intervention. In the pilot, the provision of all text messages to each patient received a high degree of focus because the sample size was small. At a larger scale, it is possible that technical issues with the intervention could have been missed, thus lowering the quality of the intervention itself; we were unable to survey patients in this regard in this study. These biases, as well as others, may explain the discrepancy in results between this randomized clinical trial and our prior pilot study. They serve as a cautionary tale to contextualize pilot study results in general and speak to the importance of evaluating an intervention in a randomized setting.

Several studies have evaluated text message–based interventions to improve colonoscopy adherence, with varying results. For example, in a German multicenter randomized clinical trial, Walter et al^15^ found that a text message reminder system significantly improved colonoscopy bowel preparation compared with usual care controls. Similarly, a 2020 study in 3 clinics in Hong Kong, China, by Lam et al^16^ found that text message reminders in the 7 to 10 days prior to outpatient colonoscopy reduced nonadherence rates, and a 2020 study by Rogers et al^17^ demonstrated improved appointment attendance rates with an interactive text messaging program among patients receiving care at Veterans Health Administration centers. By contrast, a 2018 prospective randomized clinical trial by Patel et al^18^ found no benefit in terms of bowel preparation quality associated with a text message intervention compared with usual care. A 2015 study in Korea by Lee et al^19^ also showed similar Boston Bowel Preparation Scale scores in patients receiving a texting intervention compared with telephone reminders. The heterogeneity in these findings highlights several underlying challenges in affecting behavioral change. Unlike a therapeutic trial in which interventions are typically standardized across broad cohorts, behavior change interventions must be tailored to unique contexts with different clinical processes. For example, a smartphone application may work well for health-literate populations, but not for underserved groups in which smartphone prevalence is lower. Similarly, different clinics may have different baseline processes for encouraging adherence to colonoscopy instructions, such as telephone calls and preprocedure visits. It could be hypothesized that centers with minimal resources to support adherence to outpatient colonoscopy instructions may benefit more from an automated text messaging program. Additionally, it is more difficult to demonstrate the benefit of an intervention on appointment adherence in settings in which the baseline colonoscopy nonattendance rate is relatively low. This may be the case with the present study, in which the control arm nonattendance or cancellation rate was 19.0%, compared with the study by Rogers et al^17^ study, in which the control nonattendance rate was 49.2%. These diverse considerations must be evaluated on a case-by-case basis, and it is likely that a 1-size-fits-all intervention to improve adherence and attendance for colonoscopy appointments may not currently exist. Instead, there should be a focus on behavior change frameworks to design interventions, wherein discrete component constructs are investigated and addressed for a patient population.^20,21^ This approach would emphasize processes to design a tailored intervention rather than replicating a package and may lead to improved outcomes.

Despite the lack of clinical benefit with use of a text message intervention in this study, it is important to highlight that the intervention did not diminish the quality in outcomes attributable to usual care. Although this prospect requires further study, it would be interesting to compare the utility of an automated text message intervention alone, rather than as a supplement to usual care. This could help determine if automated text messages could take the place of nursing telephone calls to all patients scheduled for colonoscopy, on which our institution currently relies. Nursing telephone calls represent a labor-intensive and time-consuming process. By contrast, automated scheduled text messages come at minimal cost and do not require any staffing overhead to directly interface with patients. We also note that enrollment in text messages likely does not require telephone calls or in-person confirmation, as we observed similar results with an opt-out model performed exclusively over text. Given these considerations, a texting program may confer a resource efficiency or cost benefit without compromising colonoscopy screening rates or bowel preparation quality.

This study was strengthened by its prospective, randomized design. Furthermore, embedding the intervention in a naturalized clinical setting with waiver of informed consent allowed us to reach out to all eligible patients and test the intervention in a real-world, pragmatic environment. Another strength of this study was the high proportion of Black patients included (>55%), as this group has historically poorer colorectal cancer screening outcomes and may stand to benefit most from novel interventions.

### Limitations

There are several limitations in this study. First, we were not able to assess bowel preparation quality using a validated scoring system owing to prevalent institutional practices. Second, we do not provide a detailed assessment of patients who received prior colonoscopy. It is possible, for example, that patients undergoing colonoscopy for the first time could benefit more from a supplemental intervention compared with patients who are experienced with colonoscopy. Third, the manner of enrollment was changed halfway through the study, which may have introduced unanticipated heterogeneity in the response to the intervention.

## Conclusions

In this large randomized clinical trial among patients undergoing outpatient colonoscopy, we did not observe differences in screening adherence with use of a tailored text message intervention. As these results are in contrast to a prior pilot study, this study underscores the importance of evaluating an intervention in a randomized setting rather than scaling systems based on pilot results. Incorporating behavior change principles into the design of an intervention may increase the likelihood of affecting outcomes that hinge on behavior change.

## Notes

Supplement 1. **Trial Protocol and Statistical Analysis Plan**

Supplement 2. **Data Sharing Statement**
