# Supplementary material for: Effect of Text Messaging on Bowel Preparation and Appointment Attendance for Outpatient Colonoscopy: A Randomized Clinical Trial
Source: JAMA Netw Open. 2021 Jan 25;4(1):e2034553. doi: 10.1001/jamanetworkopen.2020.34553 (PMC7835713; doi:10.1001/jamanetworkopen.2020.34553)
Supplement: Supplement 1. — Trial Protocol and Statistical Analysis Plan [file jamanetwopen-e2034553-s001.pdf]

**Supplement**

# Automated Text Message Navigation to Improve Outpatient Colonoscopy Show Rate and Bowel Preparation

This supplement provides additional information about the work. It contains the following items:


Initial Protocol …………………………………………………………………………………………. 1

Final Protocol …………………………………………………………………………………………. 10

Summary of protocol changes …………………………………………………………………………. 20

Original statistical analysis plan ……………………………………………………………………… 21

Final statistical analysis plan …………………………………………………………………………. 22

Summary of statistical analysis plan changes ………………………………………………………… 23

Appendix A: Intervention Text Messaging Schedule and Content …………………………………. 24

Appendix B: Telephone Consent Script ………………………………………………………………. 25

# Initial Protocol

**Automated Text Message Navigation to Improve Outpatient Colonoscopy Show Rate and Bowel Preparation**

**Brief Description**

Outpatient colonoscopy adherence is negatively impacted by poor communication and challenges with bowel preparation. We plan to perform a randomized controlled trial at the Pennsylvania Presbyterian Medical Center to (1) provide text message-based educational and reminder messages to patients regarding a scheduled colonoscopy, and (2) evaluate the impact of the texting intervention on colonoscopy show rate and bowel preparation.

**Protocol**

Abstract

Colonoscopy is an effective screening technique for colorectal cancer (CRC) prevention, but many patients either do not show up or have poor bowel preparation for the procedure. We plan to evaluate the impact and feasibility of a text message-based program to provide patients with timely educational and reminder messages regarding their upcoming colonoscopy and bowel preparation process. In this pragmatic randomized controlled trial, we aim to (1) to provide text message-based educational and reminder messages to patients or support partners regarding a scheduled colonoscopy, and (2) to evaluate the impact of the texting intervention on colonoscopy show rate and bowel preparation. We will include patients who are scheduled for outpatient colonoscopy at the Pennsylvania Presbyterian Medical Center. After enrollment, patients will be randomized 1:1 to usual care (arm 1) or the text message-based intervention (arm 2, one week in duration). We will measure colonoscopy show rate with good/excellent bowel preparation as the primary outcome.

**Objectives**

Overall objectives

The specific aims of the study are to (1) to provide text message-based educational and reminder messages to patients or support partners regarding a scheduled colonoscopy, and (2) to evaluate the impact of the texting intervention on colonoscopy show rate and bowel preparation.

**Primary outcome variable(s)**

The primary outcome of the study will be colonoscopy show rate with good/excellent bowel preparation (binary).

**Secondary outcome variable(s)**

Secondary outcomes of the study will include colonoscopy show rate, bowel preparation quality (poor, fair, good, excellent), colonoscopy cancellation rate, timing of advance cancellation notification (days), and colonoscopy appointment no-show rate.

**Background**

Colorectal cancer (CRC) is the second leading cause of cancer death in the US, yet there are effective screening and treatment strategies that allow for early detection and treatment. CRC screening is recommended for all individuals aged 50-75, which could include stool testing or colonoscopy, but national rates are still suboptimal at 59-65%. Colonoscopy is an essential component of CRC screening, as it is also required if stool testing is positive. However, colonoscopy requires a complex process to identify an escort, purchase the preparation, take a day off from work, adhere to a clear liquid diet, and complete the split-dose preparation as recommended. This results in a significant no-show and cancellation rate, along with suboptimal preparation quality, which can lead to non-adherence and incomplete screening.

Current approaches to engaging patients include having nurses call patients before the procedure or patient navigators. However, it is often difficult to get patients on the phone, and these interventions can be costly, making it less scalable for clinical practices. Other interventions such as videos or mobile apps have been limited by poor user experience or limited engagement with the patient. There is an opportunity to leverage an automated text message navigation intervention using the Way to Health (WTH) platform to improve patient engagement prior to colonoscopy completion. The WTH platform is a Penn Medicine platform that is hosted on site at the University of Pennsylvania. The platform allows custom text messages to automatically be sent to patients, in addition to bidirectional message capabilities. WTH is protected by a secure firewall and is a HIPAA compliant platform.

In the past year, our team conducted a quality improvement pilot initiative using WTH that tested the feasibility and impact of a one-week text messaging protocol for patients who were scheduled for outpatient colonoscopy. The text messages sent to patients contained information about the preparation process and instructions, expectations about the procedure, and reminders about location and timing. Among the 21 patients enrolled in the pilot, we found high user acceptability and higher colonoscopy show rates as compared to baseline values at PPMC (90% versus ~50%). As such we believe that the texting intervention is feasible for testing in the context of a randomized controlled trial.

**Study Design**

Design

We will perform a pragmatic randomized controlled trial evaluating the impact of the text message-based intervention (arm 2) as compared to usual care alone (arm 1). Patient enrollment will be performed with assistance from the call center at the Pennsylvania Presbyterian Medical Center (PPMC), and/or through phone calls from a clinical research coordinator. After enrollment, patients will be randomized 1:1 to the arms listed above through the Way to Health platform. Although patients in arm 2 may be enrolled weeks or months in advance of their colonoscopy, they will only receive intervention text messages in the 7 days prior to the scheduled colonoscopy. Usual care consists of a phone call from the PPMC endoscopy staff in the week prior to colonoscopy, if the patient is able to be reached. Patients are also given the endoscopy phone number, and may call to speak to staff if they have specific questions about their colonoscopy or need to reschedule. A detailed outline of study design can be found in the procedures sections.

**Study duration**

The duration of participation for patients in the intervention will be from the time of enrollment to the date of scheduled colonoscopy. However, the patient will only receive intervention text messages in the week prior to the scheduled colonoscopy. We will plan to recruit patients from November 2018 through January 2019. We estimate that the study will be completed by March 2019.

**Resources necessary for human research protection**

Describe research staff and justify that the staff are adequate in number and qualifications to conduct the research. Describe how you will ensure that all staff assisting with the research are adequately informed about the protocol and their research related duties. Please allow adequate time for the researchers to conduct and complete the research. Please confirm that there are adequate facilities for the research.

The project will take place at the Pennsylvania Presbyterian Medical Center (PPMC) at the University of Pennsylvania (UPENN). The team includes investigators experienced in clinical medicine, health behavior interventions, clinical trials, behavioral economics, and program evaluation. This study will be led by Dr. Shivan Mehta, MD, MBA, MSHP, Assistant Professor of Medicine and Associate Chief Innovation Officer for Penn Medicine. This study will be supported on a secure web portal on the Way to Health platform, modified to the specifications of this study. Additional study staff include a gastroenterology fellow, medical student, key PPMC endoscopy staff, and quality improvement specialists. Regular group meetings will be held at pivotal points in the trial in order to ensure compliance with protocol and research-related duties.

**Characteristics of the Study Population**

Target population

Eligibility Criteria: Patients will be eligible for study inclusion if they are age 18-85 years and are scheduled for outpatient colonoscopy to be completed at the PPMC outpatient endoscopy center. We will exclude patients if there are fewer than 10 days from time of scheduling to the scheduled date of colonoscopy. The target enrollment sample size is 400 patients—200 in arm 1 (usual care) and 200 in arm 2 (usual care plus texting intervention). This number is based on sample size calculations using data from a previous pilot, which suggests that a total sample size of 200 patients will have greater than 80% power to detect a 15% difference in the primary outcome.

Subjects enrolled by Penn Researchers

400

Subjects enrolled by Collaborating Researchers

0

**Accrual**

Participants in the study will be planned for outpatient colonoscopy at the Pennsylvania Presbyterian Medical Center (PPMC). Once a patient is confirmed to meet eligibility criteria, patient enrollment will be performed with assistance from the call center at PPMC, and/or through phone calls from a clinical research coordinator. When patients have a physician order for a colonoscopy, the patient must contact the call center in order to schedule the colonoscopy. The call center staff have agreed to discuss the research project and enroll patients using a script to be approved by the IRB. Importantly, we are requesting a waiver of consent for several reasons: (1) the study presents no more than minimal risk of harm to participants, (2) the patients who are randomized to either arm will not be deprived of any standard care available in the status quo and thus the study will not violate their rights or welfare, (3) the study could not be practicably completed without such a waiver, as the scheduling process is handled over the phone by call center staff, and (4) requiring standard informed consent would require an additional touchpoint by the research coordinator and the consent process would preclude us from evaluating the intended intervention by introducing selection bias and altering the intervention that patients receive. After enrollment, patients will be 1:1 randomized to arm 1 or arm 2 using the Way to Health platform.

Key inclusion criteria

Eligibility Criteria: Patients between ages 18 and 85 years who are scheduled for outpatient colonoscopy at PPMC and have a cell phone

Key exclusion criteria

Exclusion Criteria: Patients will be excluded if there are fewer than 10 days between the time of enrollment and time of scheduled colonoscopy. This will ensure that patients randomized to arm 2 will have sufficient time to receive the full text message intervention. Patients will also be excluded if they do not meet all of the inclusion criteria, or if they refuse participation in text messaging.

**Procedures**

Screening – Phase 1: Patients who have a physician order for a colonoscopy will call the PPMC call center in order to schedule their procedure. At this time, the call center staff will screen the patient for eligibility (age criteria and text messaging ability) and will explain the details of the study following a pre-determined script. Patients who agree to participate in the study will have their cell phone number recorded. A list of these patients will be forward to the clinical research coordinator on a weekly basis, who will confirm patient eligibility and then manually enter patients into the WTH platform. Based on our experience with the QI pilot described above, we estimate needing to screen between 500 and 600 patients to meet our enrollment targets (400 total patients).

Randomization - Phase 2: using a random number generator in the WTH platform, patients will be 1:1 randomized to arm 1 or arm 2, until the target sample size of 200 patients per arm is reached. Randomization will be performed and recorded in WTH.

Chart review - Phase 3: after randomization and in tandem with phase 4 (below), the research coordinator will perform a chart review to obtain patient demographic and comorbidity data (hypertension, hyperlipidemia, diabetes, inflammatory bowel disease, obesity, active opiate prescription). All data will be inputted into and stored in a secured RedCap database. Only key study staff will have access to the RedCap database, which is stored on a secure firewall-protected server.

Intervention - Phase 4: Patients in arm 2 will begin receiving text messages per a pre-determined protocol starting 7 days prior to the date of scheduled colonoscopy (the text messaging protocol is attached to this application). Patients in arm 1 and arm 2 will both receive usual care, which includes (1) bowel preparation instructions that are delivered via mail or through a secure online messaging portal, (2) a phone call from the endoscopy staff in the week prior to colonoscopy, and (3) the option to call the endoscopy staff during business hours to have any questions answered on demand.

Outcomes data collection - Phase 5: After a patient's scheduled colonoscopy date has passed, the research coordinator will review the medical record to gather additional data from the endoscopy procedure. This will include the indication for the procedure, quality of bowel preparation (poor, fair, good, excellent), and completeness (cecum reached). Procedure status will also be recorded (canceled, no-show, completed).

Statistical Analysis and Manuscript Preparation - Phase 6: The statistical analysis (detailed below) will commence after completion of outcomes ascertainment for the entire cohort.

**Analysis Plan**

Descriptive analysis will include comparison of baseline demographics and major medical comorbidities between study arms. Continuous and categorical data will be compared using the Wilcoxon Rank-sum and Chi-squared tests, respectively. An alpha threshold of 0.05 will be used for statistical significance, with two-tailed testing performed in all instances. For the primary analysis, we will perform a Chi-squared test of independence to compare the proportion of colonoscopies with good/excellent bowel preparation between groups. In the secondary analysis, Chi-squared tests of independence will be performed to compare colonoscopy status (show, no-show, cancelation) as well as bowel preparation quality (excellent, good, fair, poor) between groups. Finally, among canceled colonoscopies, median lead time of cancellation will be compared between groups using the Wilcoxon Rank-sum test.

**Consent**

***1. Consent Process***

**Overview**

We are requesting a waiver of informed consent for this study. The reason for our request is explained in the Waiver of Consent section. Verbal consent for text messaging will still be obtained from all participants in the study. Please see the script templates attached to this application.

**Children and Adolescents**

Not applicable. We are only enrolling subjects 18 years of age and older.

**Adult Subjects Not Competent to Give Consent**

We are requesting a waiver of informed consent for this study but patients will still need to verbally opt in to text messaging. We plan to enroll only those patients who are competent at time of enrollment to opt-in for themselves.

***2. Waiver of Consent***

**Waiver or Alteration of Informed Consent***

Waiver or alteration of required elements of consent.

**Minimal Risk***

This study is minimal risk as all participants will be receiving standard clinical care. Colonoscopy is the standard of care for colorectal cancer screening, and all patients will receive standard bowel preparation instructions in addition to optional phone communication with endoscopy staff prior to the procedure. The only research-related activity is the randomization of participants to a text messaging program that will supplement routine practice when an individual schedules a colonoscopy at our institution. Since this study is intended to promote the standard of care for colorectal cancer screening, and the receipt of text messages poses negligible risk to the patient, we believe that waiver of consent is appropriate.

**Impact on Subject Rights and Welfare***

Participation in the intervention is completely voluntary and subject rights and welfare will not be adversely affected by the waiver of authorization and consent. Participants randomized to the intervention arms still need to opt in to text messaging: Arm 1 will receive standard clinical care and Arm 2 will be enrolled into the text-messaging program in addition to standard clinical care. Placement in either arm for the purposes of research does not adversely affect the rights and welfare of the subjects, as each arm has the opportunity to engage in colorectal cancer screening, receive bowel preparation instructions, and communicate with the endoscopy staff over the phone. Randomly assigning patients to intervention and control does not alter patient's rights any differently than conducting an uncontrolled pilot study where some patients receive the intervention and some don't.  We believe it is appropriate to obtain waiver of consent because learning the impact of different modalities for improving colorectal screening has significant potential clinical value for the practice (i.e. significantly increasing the rate of successful screening and thus decreasing the rate of mortality related to nonuse of screening).

**Waiver Essential to Research***

The informed consent process itself may influence the outcome of our study, as it would require an additional phone call by the research coordinate to ask for participation. The purpose of the Way to Health text messaging program is to provide timely reminders regarding colonoscopy preparation. Obtaining waiver of consent would allow us to avoid the potential selection/volunteer bias for inclusion of patients who answer the phone and may be particularly motivated to complete colon cancer screening. Since our main objective is to understand the potential influence of the text messaging intervention on colonoscopy show rate and adequate bowel preparation quality among all patients who agree to text messaging, we believe that obtaining informed consent separately from scheduling would not allow us to evaluate the impact of the intended intervention, which is text messaging navigation offered to all patients that agree during scheduling. Additionally, participants will be recruited via verbal communication when they call our institution call center to schedule their colonoscopy. We believe the research presents no more than minimal risk of harm to subjects and involves no procedures for which written consent is normally required outside of the research context.

**Additional Information to Subjects**

Participants randomized to the intervention arms will be informed that this program is voluntary and that they can stop participating in the program (stop receiving text messages) at any time.

**Written Statement of Research***

No

**Risk / Benefit**

**Potential Study Risks**

The risks associated with this study are no more than minimal. There is the potential risk of breach of confidentiality involving medical records reviews or text messaging which will be maintained on the Way to Health platform. We will minimize this risk by using de-identified information whenever possible and by maintaining all identifiable information on a secure drive and/or in a HIPAA-compliant system (e.g. REDCap). In addition, all personnel will be held to high standards of upholding confidentiality and safeguarding patient privacy.

**Potential Study Benefits**

The benefits of this study for participants include a platform that may improve the bowel preparation experience prior to colonoscopy and increase the likelihood that a colonoscopy will not be hindered by inadequate preparation or a missed/canceled appointment. This will occur through a set of curated text messages with timely reminders as well as information with online links and a phone number to improve accessibility for questions to be answered on a timely basis. It is also possible that the benefits for some participants will be minimal. However, as mentioned, we believe the risks are also minimal. The control group is unlikely to directly benefit, as this group will continue to receive usual care. The potential public health impact of a successful intervention to improve colonoscopy show rates and bowel preparation quality is significant and could increase the chances of identifying colorectal cancer at an early stage and reduce the number of repeat colonoscopies and related costs due to inadequate bowel preparation. Information learned from this study may benefit society through a better understanding of how to effectively increase the rate of adequate colonoscopies which could increase the rate of colorectal cancer screening and reduce the rate of mortality.

**Alternatives to Participation (optional)**

**Data and Safety Monitoring**

The study is minimal risk to participants and therefore the Principal Investigators and study team will monitor the safety of this study on an ongoing basis.

**Risk / Benefit Assessment**

The risks associated with this study are no more than minimal. Better knowledge on how to increase colonoscopy show rates and improve bowel preparation quality could potentially address one of the major barriers to appropriate colorectal cancer screening, which is the second leading cause of cancer death in the US. This study is designed to test an intervention with demonstrated feasibility and successful preliminary results in a small QI pilot. For these reasons and those outlined in the above benefits section, the investigators believe that the potential benefits outweigh the risks of participating in the study.

# Final Protocol

**Automated Text Message Navigation to Improve Outpatient Colonoscopy Show Rate and Bowel Preparation**

****New changes from initial protocol notated in bold, parts removed from initial protocol notated in strikethrough**

**Brief Description**

Outpatient colonoscopy adherence is negatively impacted by poor communication and challenges with bowel preparation. We plan to perform a randomized controlled trial at the Pennsylvania Presbyterian Medical Center to (1) provide text message-based educational and reminder messages to patients regarding a scheduled colonoscopy, and (2) evaluate the impact of the texting intervention on colonoscopy show rate and bowel preparation.

**Protocol**

Abstract

Colonoscopy is an effective screening technique for colorectal cancer (CRC) prevention, but many patients either do not show up or have poor bowel preparation for the procedure. We plan to evaluate the impact and feasibility of a text message-based program to provide patients with timely educational and reminder messages regarding their upcoming colonoscopy and bowel preparation process. In this pragmatic randomized controlled trial, we aim to (1) to provide text message-based educational and reminder messages to patients or support partners regarding a scheduled colonoscopy, and (2) to evaluate the impact of the texting intervention on colonoscopy show rate and bowel preparation. We will include patients who are scheduled for outpatient colonoscopy at the Pennsylvania Presbyterian Medical Center. After enrollment, patients will be randomized 1:1 to usual care (arm 1) or the text message-based intervention (arm 2, one week in duration). We will measure colonoscopy show rate with good/excellent bowel preparation as the primary outcome.

**Objectives**

Overall objectives

The specific aims of the study are to (1) to provide text message-based educational and reminder messages to patients or support partners regarding a scheduled colonoscopy, and (2) to evaluate the impact of the texting intervention on colonoscopy show rate and bowel preparation.

**Primary outcome variable(s)**

The primary outcome of the study will be colonoscopy show rate with good/excellent bowel preparation (binary).

**Secondary outcome variable(s)**

Secondary outcomes of the study will include colonoscopy show rate, bowel preparation quality (rescue, poor, fair, **adequate,** good, excellent**; we will also collect Boston Bowel Prep Score if available [scale 0-9]**), colonoscopy cancellation rate, timing of advance cancellation notification (days), **colonoscopy reschedule rate,** and colonoscopy appointment no-show rate. **A colonoscopy "show" will be defined as a patient who attends their originally scheduled colonoscopy appointment. A "cancellation" will be defined as an appointment that is canceled at least one day prior to the originally scheduled colonoscopy appointment date. A "reschedule" will be defined as an appointment that is canceled and rescheduled (for a future date) on the same day, at least one day prior to the originally scheduled colonoscopy appointment date. Finally, as an additional secondary outcome, we will also collect the proportion of patients who opt out of the texting program in the late phase of enrollment (described below).**

**Background**

Colorectal cancer (CRC) is the second leading cause of cancer death in the US, yet there are effective screening and treatment strategies that allow for early detection and treatment. CRC screening is recommended for all individuals aged 50-75, which could include stool testing or colonoscopy, but national rates are still suboptimal at 59-65%. Colonoscopy is an essential component of CRC screening, as it is also required if stool testing is positive. However, colonoscopy requires a complex process to identify an escort, purchase the preparation, take a day off from work, adhere to a clear liquid diet, and complete the split-dose preparation as recommended. This results in a significant no-show and cancellation rate, along with suboptimal preparation quality, which can lead to non-adherence and incomplete screening.

Current approaches to engaging patients include having nurses call patients before the procedure or patient navigators. However, it is often difficult to get patients on the phone, and these interventions can be costly, making it less scalable for clinical practices. Other interventions such as videos or mobile apps have been limited by poor user experience or limited engagement with the patient. There is an opportunity to leverage an automated text message navigation intervention using the Way to Health (WTH) platform to improve patient engagement prior to colonoscopy completion. The WTH platform is a Penn Medicine platform that is hosted on site at the University of Pennsylvania. The platform allows custom text messages to automatically be sent to patients, in addition to bidirectional message capabilities. WTH is protected by a secure firewall and is a HIPAA compliant platform.

In the past year, our team conducted a quality improvement pilot initiative using WTH that tested the feasibility and impact of a one-week text messaging protocol for patients who were scheduled for outpatient colonoscopy. The text messages sent to patients contained information about the preparation process and instructions, expectations about the procedure, and reminders about location and timing. Among the 21 patients enrolled in the pilot, we found high user acceptability and higher

colonoscopy show rates as compared to baseline values at PPMC (90% versus ~50%). As such we believe that the texting intervention is feasible for testing in the context of a randomized controlled trial.

**Study Design**

Design

We will perform a pragmatic randomized controlled trial evaluating the impact of the text message-based intervention (arm 2) as compared to usual care alone (arm 1). Patient enrollment will **proceed through two pathways (early phase and late phase). In the early phase, 250 patients will be enrolled through** ~~be performed with assistance from the call center at the Pennsylvania Presbyterian Medical Center (PPMC), and~~/or through phone calls from a clinical research coordinator**, using a script pre-approved by the University of Pennsylvania Institutional Review Board**. After enrollment, patients will be randomized 1:1 to the arms listed above through the Way to Health platform. Although patients in arm 2 may be enrolled **more than one** week~~s or months~~ in advance of their colonoscopy, they will only receive intervention text messages in the 7 days prior to the scheduled colonoscopy**, in addition to two text messages at the time of enrollment explaining the texting program**. Usual care consists of a phone call from the PPMC endoscopy staff in the week prior to colonoscopy, if the patient is able to be reached. Patients are also given the endoscopy phone number, and may call to speak to staff if they have specific questions about their colonoscopy or need to reschedule. **In the late phase, an additional 500 patients will be automatically enrolled in the Way to Health system using verified cell phone numbers from the electronic medical record system, where they will be randomized in a 1:1 ratio to the study arms in variable blocks of 8 and 4. Patients in arm 2 will receive two enrollment text messages explaining the texting program and providing the opportunity to opt out (by replying with the text STOP).** A detailed outline of **the** study design can be found in the procedures sections.

**Study duration**

The duration of participation for patients in the intervention will be from the time of enrollment to the date of scheduled colonoscopy. However, the patient will only receive intervention text messages in the week prior to the scheduled colonoscopy**, with the exception of the two enrollment messages in the late phase**. We will plan to recruit patients from November 2018 through ~~January~~ **June** 2019. We estimate that the study will be completed by ~~March~~ **August** 2019.

**Resources necessary for human research protection**

Describe research staff and justify that the staff are adequate in number and qualifications to conduct the research. Describe how you will ensure that all staff assisting with the research are adequately informed about the protocol and their research related duties. Please allow adequate time for the researchers to conduct and complete the research. Please confirm that there are adequate facilities for the research.

The project will take place at the Pennsylvania Presbyterian Medical Center (PPMC) at the University of Pennsylvania (UPENN). The team includes investigators experienced in clinical medicine, health behavior interventions, clinical trials, behavioral economics, and program evaluation. This study will be led by Dr. Shivan Mehta, MD, MBA, MSHP, Assistant Professor of Medicine and Associate Chief Innovation Officer for Penn Medicine. This study will be supported on a secure web portal on the Way to Health platform, modified to the specifications of this study. Additional study staff include a gastroenterology fellow, **a clinical research coordinator, a** medical student, key PPMC endoscopy staff, and quality improvement specialists. Regular group meetings will be held at pivotal points in the trial in order to ensure compliance with protocol and research-related duties.

**Characteristics of the Study Population**

Target population

Eligibility Criteria: Patients will be eligible for study inclusion if they are age 18-85 years and are scheduled for outpatient colonoscopy to be completed at the PPMC outpatient endoscopy center. We will exclude patients if there are fewer than 1**4** ~~10~~ days from time of scheduling to the scheduled date of colonoscopy. The target enrollment sample size is **750** ~~400~~ patients— **375** ~~200~~ in arm 1 (usual care) and **375** ~~200~~ in arm 2 (usual care plus texting intervention). This number is based **in part** on sample size calculations using data from a previous pilot, which suggests that a total sample size of **750** ~~200~~ patients will have greater than 80% power to detect a **10** ~~15~~% difference in the primary outcome**. We also chose a larger sample size to account for the fact that patients may opt out of the text messages in the late phase of enrollment, which would likely decrease the effect size between arms.**

Subjects enrolled by Penn Researchers

**750** 400

Subjects enrolled by Collaborating Researchers

0

**Accrual**

Participants in the study will be planned for outpatient colonoscopy at the Pennsylvania Presbyterian Medical Center (PPMC). Once a patient is confirmed to meet eligibility criteria, patient enrollment will ~~be performed~~ **proceed based on one of two pathways (early and late phase). In the early phase (first 250 patients), enrollment will be performed** ~~with assistance from the call center at PPMC, and/or~~ through phone calls from a clinical research coordinator~~. When patients have a physician order for a colonoscopy, the patient must contact the call center in order to schedule the colonoscopy. The call center staff have agreed to discuss the research project and enroll patients using a script to be approved by the IRB. Similarly,~~ **The research coordinator will call newly scheduled patients to discuss the**

**research project and enroll patients using a script to approved by the University of Pennsylvania IRB. If patients are scheduled for colonoscopy in person while at the office, they will receive an informational flyer about the study that gives them the opportunity to contact a study team member for additional information or to enroll. It also informs them that someone from the study may contact them directly about participating. After enrollment, patients will be 1:1 randomized to arm 1 or arm 2 using the Way to Health platform.**

**In the late phase (additional 500 patients), patients with upcoming colonoscopy appointments will be screened for eligibility as per the selection criteria. As in the early phase, if patients are scheduled for colonoscopy in person while at the office, they will receive an informational flyer about the study that lets them know they may be contacted by text message about their upcoming procedure. Those who qualify will have their validated cell phone numbers imported into Way to Health, where they will subsequently be randomized. Those in the intervention arm will receive two enrollment messages describing the study and providing the opportunity to opt out (by replying with the text STOP). Of note, if a patient in the late phases chooses to opt out and not receive the texting intervention, their data will still be analyzed in the intervention group (as an intention-to-treat approach).** Importantly, we are requesting a waiver of consent for several reasons: (1) the study presents no more than minimal risk of harm to participants, (2) the patients who are randomized to either arm will not be deprived of any standard care available in the status quo and thus the study will not violate their rights or welfare, (3) the study could not be practicably completed without such a waiver, as the scheduling process is handled over the phone by call center staff, and (4) requiring standard informed consent would require an additional touchpoint by the research coordinator and the consent process would preclude us from evaluating the intended intervention by introducing selection bias and altering the intervention that patients receive.

Key inclusion criteria

Eligibility Criteria: Patients between ages 18 and 85 years who are scheduled for outpatient colonoscopy at PPMC and have a cell phone **with enabled text messaging capability.**

Key exclusion criteria

Exclusion Criteria: Patients will be excluded if there are fewer than **14** ~~10~~ days between the time of enrollment and time of scheduled colonoscopy. This will ensure **the clinical research coordinator has sufficient time to reach newly scheduled patients, and** that patients randomized to arm 2 will have sufficient time to receive the full text message intervention. Patients will also be excluded if they **are non-English speaking requiring a translator, or if they are not the primary individual receiving the text messages. Finally, patients will be excluded if they** do not meet all of the inclusion criteria, or if they refuse participation in text messaging.

**Procedures**

Screening – Phase 1: **As above, patients will be screened and enrolled through one of two pathways (early and late phase). In the early phase (first 250 patients),** ~~Patients who have a physician order for a colonoscopy will call the PPMC call center in order to schedule their procedure. At this time, the call center staff will screen the patient for eligibility (age criteria and text messaging ability) and will explain the details of the study following a pre-determined script. Patients who agree to participate in the study will have their cell phone number recorded. A list of these patients will be forward to the clinical research coordinator on a weekly regular basis, who will confirm patient eligibility and then manually enter patients into the WTH platform for randomization. Alternatively,~~ **a PPMC practice manager will forward a list of scheduled colonoscopies to the clinical research coordinator (CRC) on a regular basis. If patients are scheduled for colonoscopy in person while at the office, they will receive an informational flyer about the study that gives them the opportunity to contact a study team member for additional information or to enroll. It also informs them that someone from the study may contact them directly about participating. The CRC will screen** ~~for~~ **patients for eligibility and make up to 3 phone call attempts to reach the patient. Once on the phone, the CRC will explain the details of the study using a script approved by the IRB and enter patients directly into the WTH platform for randomization. In the late phase (additional 500 patients), patients will be screened for eligibility through the same process as in the early phase. If they are scheduled for colonoscopy in person while at the office, they will also receive an informational flyer about the study that lets them know they may be contacted by text message about their upcoming procedure. For eligible patients, the CRC will then manually enter the validated patient cell phone number from the electronic medical record system into WTH. Randomization will then occur as described below, and intervention arm patients will receive two enrollment messages. These messages will describe the texting program and provide the opportunity for patients to opt out (by texting the word STOP). Of note, phone numbers will be confirmed to represent cell phones by using a publicly-available lookup utility. Importantly, patients who opt out will still be included in the intervention arm as an intention-to-treat analysis.** Based on our experience with the QI pilot described above, we estimate needing to screen ~~between 500 and~~ **approximately 1,000** ~~600~~ patients to meet our enrollment targets (**750** ~~400~~ total patients).

Randomization - Phase 2: **In the early phase,** using a random number generator in the WTH platform, patients will be 1:1 randomized to arm 1 or arm 2**. Beginning in the late phase of enrollment, patients** ~~,~~ ~~until the target~~ **will be randomized to arm 1 or arm 2 in a 1:1 ratio in variable blocks of 8 and 4 using the WTH platform, until the target sample size of 375 patients per arm is reached.** ~~sample size of 200 patients per arm is reached. Randomization will be performed and recorded in WTH.~~

Chart review - Phase 3: after randomization and in tandem with phase 4 (below), the research coordinator will perform a chart review to obtain patient demographic and comorbidity data (hypertension, hyperlipidemia, diabetes, inflammatory bowel disease, obesity, active opiate prescription). **We will also collect data on the number of patients opting out in the late phase of enrollment, as well as data on nursing phone calls which are performed as a component of usual care**

**(not called, called but could not reach patient, called and spoke with patient).** All data will be inputted into and stored in a secured RedCap database. Only key study staff will have access to the RedCap database, which is stored on a secure firewall-protected server.

Intervention - Phase 4: Patients in arm 2 will begin receiving text messages per a pre-determined protocol starting 7 days prior to the date of scheduled colonoscopy (the text messaging protocol is attached to this application). **Of note, if a patient in arm 2 cancels or reschedules their colonoscopy after randomization, they will not receive any additional protocol text messages as part of this trial.** Patients in arm 1 and arm 2 will both receive usual care, which includes (1) bowel preparation instructions that are delivered via mail or through a secure online messaging portal, (2) a phone call from the endoscopy staff in the week prior to colonoscopy, and (3) the option to call the endoscopy staff during business hours to have any questions answered on demand.

Outcomes data collection - Phase 5: After a patient's scheduled colonoscopy date has passed, the research coordinator will review the medical record to gather additional data from the endoscopy procedure. This will include the indication for the procedure, quality of bowel preparation (**rescue,** poor, fair, **adequate,** good, excellent**; Boston Bowel Prep Score will also be collected if recorded**), and completeness (cecum reached). Procedure status will also be recorded (canceled, no-show, **rescheduled,** completed). **If the procedure was canceled, the cancellation lead time (in days) prior to scheduled colonoscopy will be recorded.**

Statistical Analysis and Manuscript Preparation - Phase 6: The statistical analysis (detailed below) will commence after completion of outcomes ascertainment for the entire cohort.

**Analysis Plan**

Descriptive analysis will include comparison of baseline demographics and major medical comorbidities between study arms. Continuous and categorical data will be compared using the Wilcoxon Rank-sum and Chi-squared tests, respectively. An alpha threshold of 0.05 will be used for statistical significance, with two-tailed testing performed in all instances. For the primary analysis, we will perform a Chi-squared test of independence to compare the proportion of colonoscopies with good/excellent bowel preparation between groups. In the secondary analysis, Chi-squared tests of independence will be performed to compare colonoscopy status (show, no-show, **reschedule,** cancelation) as well as bowel preparation quality (excellent, good, **adequate,** fair, poor) between groups. ~~Finally, a~~**A**mong canceled colonoscopies, median lead time of cancellation will be compared between groups using the Wilcoxon Rank-sum test. **Finally, for both primary and secondary analyses, we will consider multivariable regression modeling to adjust for imbalances in possible confounders, if present despite randomization.**

**Consent**

***1. Consent Process***

**Overview**

We are requesting a waiver of informed consent for this study. The reason for our request is explained in the Waiver of Consent section. Verbal consent for text messaging will still be obtained from all participants in the study. Please see the script templates attached to this application.

**Children and Adolescents**

Not applicable. We are only enrolling subjects 18 years of age and older.

**Adult Subjects Not Competent to Give Consent**

We are requesting a waiver of informed consent for this study but patients will still need to verbally opt in to text messaging. We plan to enroll only those patients who are competent at time of enrollment to opt-in for themselves.

***2. Waiver of Consent***

**Waiver or Alteration of Informed Consent***

Waiver or alteration of required elements of consent.

**Minimal Risk***

This study is minimal risk as all participants will be receiving standard clinical care. Colonoscopy is the standard of care for colorectal cancer screening, and all patients will receive standard bowel preparation instructions in addition to optional phone communication with endoscopy staff prior to the procedure. The only research-related activity is the randomization of participants to a text messaging program that will supplement routine practice when an individual schedules a colonoscopy at our institution. Since this study is intended to promote the standard of care for colorectal cancer screening, and the receipt of text messages poses negligible risk to the patient, we believe that waiver of consent is appropriate.

**Impact on Subject Rights and Welfare***

Participation in the intervention is completely voluntary and subject rights and welfare will not be adversely affected by the waiver of authorization and consent. Participants randomized to the intervention arms still need to opt in to text messaging: Arm 1 will receive standard clinical care and Arm 2 will be enrolled into the text-messaging program in addition to standard clinical care. Placement in either arm for the purposes of research does not adversely affect the rights and welfare of the subjects, as each arm has the opportunity to engage in colorectal cancer screening, receive bowel preparation instructions, and communicate with the endoscopy staff over the phone. Randomly assigning patients to intervention and control does not alter patient's rights any differently than conducting an uncontrolled pilot study where some patients receive the intervention and some don't.  We believe it is appropriate

to obtain waiver of consent because learning the impact of different modalities for improving colorectal screening has significant potential clinical value for the practice (i.e. significantly increasing the rate of successful screening and thus decreasing the rate of mortality related to nonuse of screening).

**Waiver Essential to Research***

The informed consent process itself may influence the outcome of our study, as it would require an additional phone call by the research coordinate to ask for participation. The purpose of the Way to Health text messaging program is to provide timely reminders regarding colonoscopy preparation. Obtaining waiver of consent would allow us to avoid the potential selection/volunteer bias for inclusion of patients who answer the phone and may be particularly motivated to complete colon cancer screening. Since our main objective is to understand the potential influence of the text messaging intervention on colonoscopy show rate and adequate bowel preparation quality among all patients who agree to text messaging, we believe that obtaining informed consent separately from scheduling would not allow us to evaluate the impact of the intended intervention, which is text messaging navigation offered to all patients that agree during scheduling. Additionally, participants will be recruited via verbal communication when they call our institution call center to schedule their colonoscopy. We believe the research presents no more than minimal risk of harm to subjects and involves no procedures for which written consent is normally required outside of the research context.

**Additional Information to Subjects**

Participants randomized to the intervention arms will be informed that this program is voluntary and that they can stop participating in the program (stop receiving text messages) at any time.

**Written Statement of Research***

No

**Risk / Benefit**

**Potential Study Risks**

The risks associated with this study are no more than minimal. There is the potential risk of breach of confidentiality involving medical records reviews or text messaging which will be maintained on the Way to Health platform. We will minimize this risk by using de-identified information whenever possible and by maintaining all identifiable information on a secure drive and/or in a HIPAA-compliant system (e.g. REDCap). In addition, all personnel will be held to high standards of upholding confidentiality and safeguarding patient privacy.

**Potential Study Benefits**

The benefits of this study for participants include a platform that may improve the bowel preparation experience prior to colonoscopy and increase the likelihood that a colonoscopy will not be hindered by inadequate preparation or a missed/canceled appointment. This will occur through a set of curated text messages with timely reminders as well as information with online links and a phone number to improve accessibility for questions to be answered on a timely basis. It is also possible that the benefits for some participants will be minimal. However, as mentioned, we believe the risks are also minimal. The control group is unlikely to directly benefit, as this group will continue to receive usual care. The potential public health impact of a successful intervention to improve colonoscopy show rates and bowel preparation quality is significant and could increase the chances of identifying colorectal cancer at an early stage and reduce the number of repeat colonoscopies and related costs due to inadequate bowel preparation. Information learned from this study may benefit society through a better understanding of how to effectively increase the rate of adequate colonoscopies which could increase the rate of colorectal cancer screening and reduce the rate of mortality.

**Alternatives to Participation (optional)**

**Data and Safety Monitoring**

The study is minimal risk to participants and therefore the Principal Investigators and study team will monitor the safety of this study on an ongoing basis.

**Risk / Benefit Assessment**

The risks associated with this study are no more than minimal. Better knowledge on how to increase colonoscopy show rates and improve bowel preparation quality could potentially address one of the major barriers to appropriate colorectal cancer screening, which is the second leading cause of cancer death in the US. This study is designed to test an intervention with demonstrated feasibility and successful preliminary results in a small QI pilot. For these reasons and those outlined in the above benefits section, the investigators believe that the potential benefits outweigh the risks of participating in the study.

## Summary of Protocol Changes

**Protocol:** Automated Text Message Navigation to Improve Outpatient Colonoscopy Show Rate and Bowel Preparation
**University of Pennsylvania Principal Investigator:** Shivan Mehta, MD

| Date of Submission | Description of Modification | Rationale for Modification | Approval date |
|---|---|---|---|
| 9/11/18 | Initial submission | | 10/8/2018 |
| 10/22/18 | Modification:<br>- add staff (CRC)<br>- add call center script | | 10/23/2018 |
| 11/14/18 | Modification:<br>- add recruitment by CRC & call script<br>- change exclusion (from 10 to 14 days) | | 11/15/2018 |
| 12/19/18 | Modification:<br>- add patient recruitment flyer<br>- modify CRC call script | | 12/19/2018 |
| 1/2/2019 | Modification:<br>- modify CRC call script<br>- modify “welcome text” message for pts in intervention arm<br>- modify inclusion/exclusion criteria<br>- add description of procedure if arm 2 patient cancels or reschedules colonoscopy<br>- add lead time of cancellation as an outcome measure (in days) | | |
| 4/4/2019 | Modification:<br>- modify sample size target<br>- distinguish early and late phase enrollment | | |
| | | | |

# Initial Statistical Analysis Plan

The target enrollment sample size is 400 patients—200 in arm 1 (usual care) and 200 in arm 2 (usual care plus texting intervention). This number is based on sample size calculations using data from a previous pilot, which suggests that a total sample size of 200 patients will have greater than 80% power to detect a 15% difference in the primary outcome.

Descriptive analysis will include comparison of baseline demographics and major medical comorbidities between study arms. Continuous and categorical data will be compared using the Wilcoxon Rank-sum and Chi-squared tests, respectively. An alpha threshold of 0.05 will be used for statistical significance, with two-tailed testing performed in all instances. For the primary analysis, we will perform a Chi-squared test of independence to compare the proportion of colonoscopies with good/excellent bowel preparation between groups. In the secondary analysis, Chi-squared tests of independence will be performed to compare colonoscopy status (show, no-show, cancelation) as well as bowel preparation quality (excellent, good, fair, poor) between groups. Finally, among canceled colonoscopies, median lead time of cancellation will be compared between groups using the Wilcoxon Rank-sum test.

# Final Statistical Analysis Plan

**New changes from initial protocol notated in bold, parts removed from initial protocol notated in strikethrough**

The target enrollment sample size is **750** ~~400~~ patients—**375** ~~200~~ in arm 1 (usual care) and **375** ~~200~~ in arm 2 (usual care plus texting intervention). This number is based **in part** on sample size calculations using data from a previous pilot, which suggests that a total sample size of **750** ~~200~~ patients will have greater than 80% power to detect a **10** ~~15~~% difference in the primary outcome**. We also chose a larger sample size to account for the fact that some patients enrolled in arm 2 may opt out of receiving text messages, which would likely decrease the effect size between arms.**

Descriptive analysis will include comparison of baseline demographics and major medical comorbidities between study arms. Continuous and categorical data will be compared using the Wilcoxon Rank-sum and Chi-squared tests, respectively. An alpha threshold of 0.05 will be used for statistical significance, with two-tailed testing performed in all instances. For the primary analysis, we will perform a Chi-squared test of independence to compare the proportion of colonoscopies with good/excellent bowel preparation between groups. In the secondary analysis, Chi-squared tests of independence will be performed to compare colonoscopy status (show, no-show, **reschedule,** cancelation) as well as bowel preparation quality (excellent, good, **adequate,** fair, poor) between groups. ~~Finally, a~~**A**mong canceled colonoscopies, median lead time of cancellation will be compared between groups using the Wilcoxon Rank-sum test. **Finally, for both primary and secondary analyses, we will consider multivariable regression modeling to adjust for imbalances in possible confounders, if present despite randomization.**

## Summary of Statistical Analysis Plan Modifications

- Sample size adjusted
- Additional details regarding secondary outcomes
- Added possibility of regression analyses if imbalances in potential confounders are noted

# Appendix A: Intervention Text Messaging Schedule and Content

| Timing | Message |
| --- | --- |
| Upon enrollment | Congrats <name> on scheduling your colonoscopy on <date> with <provider>! We would like to offer you a texting program to guide you through the process. Just so you have it, here is an online link to the prep instructions: http://bit.ly/2nHjpbK |
| Day -7: 8AM | Hi <name>, only one week before your colonoscopy! We will be in touch this week to guide you through the process. If you take any blood thinners (like warfarin), make sure you have discussed this with your prescribing doctor. Some patients may need to stop these before the procedure. |
| Day -6: 8AM | Hello, <name>! Hopefully you received a paper copy of your colonoscopy prep instructions. Just in case, here is a link to the same instructions online: http://bit.ly/2nHjpbK |
| Day -5: 8AM | Hello <name>, don't forget to pick up your prep materials from the local pharmacy, which includes MiraLAX, Dulcolax, and Gatorade (or Crystal Light or Pedialyte if you have diabetes). This info is in the instructions at this link: http://bit.ly/2nHjpbK |
| Day -4: 8AM | Good morning, <name>! Only 4 days to go until your procedure! If you have any questions about the colonoscopy, please call 215-662-9131 between 8AM and 6PM. |
| Day -3: 8AM | Hi <name>, make sure you have someone to take you home after your colonoscopy. Plan to arrive one hour before your procedure time. Penn Endoscopy is located at the CUPP Building, 51 N. 39th St., Phila. PA. Here is a link: https://bit.ly/2rWQRwO |
| Day -2: 8AM | <name>, your colonoscopy is in 2 days! Today, avoid high-fiber foods like fruits, vegetables, and seeds. Starting tomorrow morning, you should have only clear liquids until your procedure is complete. |
| Day -1: 8AM | <name>, you have already come so far! Continue a clear liquid diet today (liquids you can see through with light colors). Remember, no solid foods! The next step will be to take 4 Dulcolax pills at 4PM. |
| Day -1: 4PM | It is time to take the 4 Dulcolax pills and mix the MiraLAX with the Gatorade (or Crystal Light or Pedialyte). At 5PM, you should start drinking the first half gallon of your prep. If you feel nauseated, you can always slow down to help tolerate it. You can do it! |
| Day -1: 6PM | Good job! Start drinking the second half gallon of the prep 6 hours before your scheduled arrival time. Try to drink every last drop to get the best prep you possibly can! |

# Appendix B: Telephone Consent Script

**Caller:** Our department is conducting a trial to improve the colonoscopy preparation experience for patients by sending a series of eight text messages in the week prior to the procedure. The messages contain information and reminders about the preparation process. Participation is completely voluntary. If you agree, you will randomly be selected to either receive the text messages or not.

Would you be willing to participate in this research study?

[If no, thank for time]

[If yes, continue]

I just want to confirm that we have the correct cell phone number for you… (confirm number)

Do you have any questions regarding your participation?

[If no, thank for time]

[If yes, address questions, and thank for time]
