# Supplementary material for: Effect of Text Messaging on Bowel Preparation and Appointment Attendance for Outpatient Colonoscopy: A Randomized Clinical Trial
Source: JAMA Netw Open. 2021 Jan 25;4(1):e2034553. doi: 10.1001/jamanetworkopen.2020.34553 (PMC7835713; doi:10.1001/jamanetworkopen.2020.34553)
Supplement: Supplement 2. — Data Sharing Statement [file jamanetwopen-e2034553-s002.pdf]

Data Sharing Statement

Mahmud. Effect of Text Messaging on Bowel Preparation and Appointment Attendance for Outpatient Colonoscopy. *JAMA Netw Open*. Published January 25, 2021. doi:10.1001/jamanetworkopen.2020.34553

Data
Data available: No
